# Analysis of genomic ancestry and characterization of a new variant in MPS type VII

**DOI:** 10.1186/s13023-025-03593-8

**Published:** 2025-04-25

**Authors:** Andreza Juliana Moreira da Costa, Isabel Cristina Neves de Souza, Raimunda Helena Feio, Laurent Ketlen Leão Viana, Mislene Cisz, Célio Luiz Rafaelli, Franciele Barbosa Trapp, Maira Graeff Burin, Kristiane Michelin-Tirelli, Ana Carolina Brusius-Facchin, Alice Brinckmann Oliveira Netto, André Salim Khayat, Ney Pereira Carneiro dos Santos, Roberto Giugliani, Luiz Carlos Santana-da-Silva

**Affiliations:** 1 Laboratory of Inborn Errors of Metabolism, Institute of Biological Sciences, Federal University of Pará, Belém, Brazil; 2 Oncology Research Center, Hospital Universitário João de Barros Barreto, Federal University of Pará, Belém, Pará Brazil; 3 Graduate Program in Genetics and Molecular Biology, Institute of Biological Sciences, Federal University of Pará, Belém, Pará Brazil; 4 Institute of Biological Sciences, Hospital Universitário Bettina Ferro de Souza, Federal University of Pará, Belém, Pará Brazil; 5 Graduate Program in Pharmacology and Biochemistry, Institute of Biological Sciences, Federal University of Pará, Belém, Pará Brazil; 6 Center for Comprehensive Care and Training in Rare Diseases, Casa dos Raros, Porto Alegre, Brazil; 7 Medical Genetics Service, Hospital de Clínicas de Porto Alegre, Porto Alegre, Rio Grande do Sul Brazil; 8 Graduate Program in Genetics and Molecular Biology, Federal University of Rio Grande do Sul, Porto Alegre, Rio Grande do Sul Brazil; 9 Laboratory Biodiscovery, Hospital de Clínicas de Porto Alegre, Porto Alegre, Rio Grande do Sul Brazil; 10 Dasa Genômica, Porto Alegre, Rio Grande do Sul Brazil; 11 Instituto Nacional de Genética Médica Populacional-INAGEMP, CNPQ, Porto Alegre, Rio Grande do Sul Brazil

**Keywords:** Ancestry, INDELs markers, MPS VII, Gene expression

## Abstract

**Background:**

Mucopolysaccharidosis (MPS) type VII is a storage disorder of autosomal recessive origin that is caused by a deficiency in a lysosomal enzyme that results in the accumulation of glycosaminoglycans and causes secondary metabolic pathway problems. It has systemic symptoms that mainly include progressive skeletal dysplasia, cardiovascular manifestations, hepatosplenomegaly, coarse facies, and many other manifestations, and cognitive decline is observed in most cases. A significant proportion of patients may present with foetal hydrops. Allelic variations in specific ethnic groups explain the higher incidence in some groups due to founder effects and/or endogamy. In Brazil, the most common variant is p.Leu176Phe. This study aimed to investigate *GUSB* gene expression in a patient with MPS VII with a new mutation (p.Leu292Pro). Additionally, this study investigated the ancestry of 5 patients with MPS VII from Brazil to understand the Amerindian, African, and European contributions.

**Results:**

The analysis revealed varying proportions of ancestry markers in the sample of patients with MPS VII. The European contribution was more prominent and significantly different (*p* = 0.0031) from the African contribution. Relative expression analysis by the 2^−ΔCT^ method revealed greater expression of the *GUSB g*ene in the patient with MPS VII than in the control group (CG). However, some samples from the CG group presented higher expression than did the samples from the patient with the new mutation. Relative to the comparison among threshold cycles, 2/20 samples presented significantly different CT values for the patient with MPS VII when the numbers of amplification cycles were compared. The parents of the patient also presented different values (*p* < 0.05) for the amplification cycles. The in silico prediction of the new variant indicated that it affects function by modifying a highly conserved region.

**Conclusions:**

The p.Leu176Phe mutation may have originated in Europe, as suggested in this study. There is a discrepancy between the mRNA levels of *GUSB* and the amount of beta-glucuronidase synthesized. The expression of the *GUSB* gene variant from the patient with MPS VII was within the range of the control group’s distribution in this study. The p.Leu292Pro mutation is pathogenic, but its impact on the MPS VII phenotype still needs to be fully elucidated.

## Background

Mucopolysaccharidosis type VII (MPS VII), also called Sly Syndrome, is caused by an inherent error of metabolism and belongs to a group of lysosomal storage diseases. The deficiency of the β-glucuronidase enzyme (EC 3.2.1.31) provokes partial degradation or does not allow the metabolism of glycosaminoglycans (GAGs), specifically dermatan sulfate (DS), heparan sulfate (HS), and chondroitin sulfate (C4S and C6S), resulting in the accumulation of GAGs in different tissues [1, 2]. The accumulation of metabolites in lysosomes can disrupt the processing of GAGs and hinder storage mechanisms, resulting in complications such as vesicle trafficking, oxidative stress, signalling pathways, and inflammation [3]. MPS VII has an autosomal recessive inheritance pattern and occurs due to the occurrence of variants in the *GUSB* gene (OMIM *611499).

The *GUSB* gene, which encodes the β-glucuronidase protein, is located in the q11.21 region of chromosome 7, is 12 kb in size, and has 11 introns, 12 exons of various sizes, and 14 transcripts. There are 71 pathogenic variants identified in the *GUSB* gene available for public access (http://www.hgmd.cf.ac.uk/ac/gene.php?gene=GUSB, accessed on June 12, 2024). These allelic variants are responsible for the high degree of clinical heterogeneity in MPS VII [4].

Disease onset may manifest during foetal development with hydrops foetalis or during infancy with hernias. Symptoms can range from skeletal abnormalities, accentuated features, cardiovascular problems, and hepatosplenomegaly. Furthermore, the presence of hydrops foetalis is a feature of MPS VII. MPS VII can cause a range of symptoms, and treatment focuses on managing specific clinical manifestations and mitigating symptoms through surgery. The treatment for MPS VII consists of enzyme replacement therapy (ERT), the introduction of a functional enzyme to normalize the GAG pathways, or haematopoietic stem cell transplantation, which has limitations because of the compatibility of the donors [5, 6].

MPS VII is a rare disorder with an estimated prevalence of 0.02–1.14 per 100,000 inhabitants worldwide [7]. According to FEDERHEN et al. [8], the estimated prevalence of MPS VII in Brazil is 0.05 per 100,000 inhabitants. The diverse spectrum of the MPS VII majority in different populations leads to the consideration of the existence of founder mutations in certain ethnicities [3]. The p.Pro408Ser variant is common in Mexico, p.Ala619Val is found in the Japanese population and has an attenuated phenotype, whereas p.Arg357Term has a severe phenotype [9].

In addition, there are a few pathogenic variants responsible for most cases of MPS VII, such as the variant NM_000181.4: c.526C>T (p.Leu176Phe) [7]. This variant is common in American, Mexican, Brazilian, Chilean, English, French, Polish, Spanish, and Turkish patients [3]. Giugliani et al. [10], through a study on the natural history of MPS VII in Brazil, reported that the p.Leu176Phe variant was present in recessive homozygosis in 12 of 13 Brazilian patients. Only one patient from northern Brazil presented compound heterozygosity including this variant and the new variant NM_000181.4: c.875T>C (p.Leu292Pro).

The *GUSB* gene is expressed in most body fluids and tissues, and variants in the gene are the main factors involved in the variability of gene expression, which plays an essential role in protein determination and is influenced by several pathways, from transcription to encoding amino acid residues to proteins.

The beta-glucuronidase monomer includes three structural domains, the first of which is essential for lysosomal targeting, comprises 651 amino acid residues, and contains a 22-residue-long signal peptide and four potential glycosylation sites. Different organisms share a conserved region that includes active sites [11].

Specific elements, such as pH and calcium ions, can influence the regulation of *GUSB* gene expression. The calcium ionophore A23187 has been shown to alter the expression of this gene. One potential modification of the final product is a variant of the amino acid sequence of beta-glucuronidase that can modify the enzyme structure and allow new chemical bonds. Additionally, posttranslational modifications to the GUSB protein can impair protein function, such as at crucial glycosylation sites for lysosomal targeting and catalytic activity. In addition, it is regulated at several levels; therefore, alterations in the DNA can modify the mRNA processing trajectory. Thus, the diversity of pathogenic or nonpathogenic variants can be essential for gene expression and protein expression [11, 12].

Considering that a single variant in the *GUSB* gene is responsible for all known cases of MPS VII in Brazil, this study aims to investigate whether there is any relationship involving a common ancestral origin. In addition, another objective is to analyse gene expression and the pathogenic effects of the new variant on the function of the GUSB protein in patients with compound heterozygosity.

## Materials and methods

The new variant in the *GUSB* gene identified in the patient with MPS VII in the present study was found to be a compound heterozygous genotype: c.526C>T (p.Leu176Phe)/c.875T>C (p.Leu292Pro) [10]; it was identified in a previous study by our group that was submitted and approved by the Research Ethics Committee of Hospital de Clínicas de Porto Alegre (HCPA)—Rio Grande do Sul (Number ° CAEE 70669417510015327). All study participants signed the informed consent form.

### Ancestry study

#### Genetic and clinical characteristics of the samples

This analysis is a retrospective cohort study in which samples for ancestry analysis consisted of 5 of the 13 patients with MPS VII whose families agreed to participate in the study. Four of them are homozygous (p.Leu176Phe/p.Leu176Phe), and one exhibits compound heterozygosity (p.Leu176Phe/p.Leu292Pro). The female patients who participated in this study were 3, 11, 14, and 22 years old, whereas the male patient was 8 years old. Among the patients’ clinical symptom histories, the doctors also observed other clinical symptoms, such as neuromotor development delay, dysostosis multiplex, and coarse facies, for all five patients. Table 1 shows each patient’s other clinical and individual characteristics.

**Table 1:** Individual characteristics of patients with MPS VII

Sample characteristics	Patient				
	1	2	3	4	5
Sex	Male	Female	Female	Female	Female
Parental consanguinity	No	Yes	Yes	Yes	No
Family history of MPS VII	No	Yes	Yes	No	No
Recurrent respiratory infections	No	Yes	Yes	Not available	Yes
Nonimmune hydrops foetalis	No	Yes	Yes	Not available	Yes
Hearing loss	No	No	Yes	Not available	Not available
Snoring	No	No	No	Not available	Yes
Genotype	C. Het	Hom	Hom	Hom	Hom

C. Het, Compound heterozygous; Hom, Homozygous

**Fig. 1:**
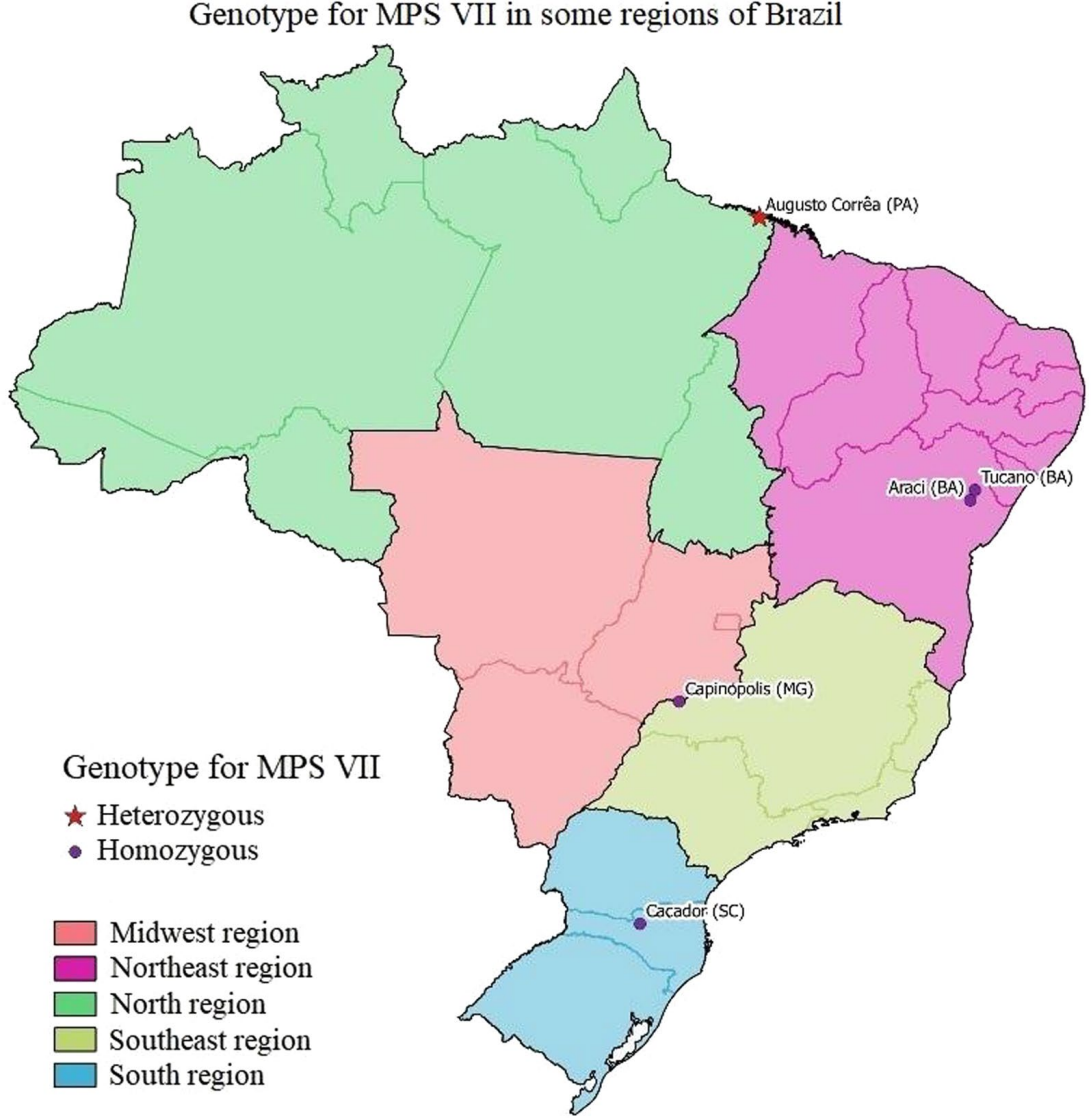
Cities of birth of the 5 patients diagnosed with MPS VII. Black filled circle: homozygosity of the c.526C>T variant (p.Leu176Phe); red filled star: compound heterozygosity c.526C>T (p.Leu176Phe)/c.875T>C (p.Leu292Pro)

The information regarding these patients is sourced from the MPS Brazil Network, with diagnoses made between 2004 and 2019. All patients received clinical, biochemical, and molecular diagnoses of MPS VII from the Medical Genetics Service—HCPA. Figure 1 shows the distribution map of patients in each city: two patients were from Araci and Tucano (State of Bahia), one was from Caçador (State of Santa Catarina), one was from Capinópolis (State of Minas Gerais), and one was from the city of Augusto Correa (State of Pará).

#### INDEL markers

Analysis by INDEL markers (which consist of deletions and insertions), which are widely used tools for mixed-race recognition, was selected to perform ancestry studies in patients. The 61 ancestry markers used were previously validated and described [13–15]. These INDEL markers can be found in the Supplementary Table of Ramos et al. [15].

The genetic profile of each patient with MPS VII was determined by analysing the sample results for the markers in the software STRUCTURE v2.3.4 [13, 16] (available at https://web.stanford.edu/group/pritchardlab/structure.html) to ascertain the relative contributions of ancestries.

Through these markers, it is possible to estimate the proportion of each ancestry that composes the Brazilian population: European, African, and Amerindian.

#### Multiplex PCR

The amplification of the DNA samples was performed using a QIAGEN Multiplex PCR kit and a Veriti® 96-well thermal cycler from Applied Biosystems™, followed by capillary electrophoresis. The DNA fragments were separated by an automatic DNA analyser using ABI PRISM 3130 equipment from Applied Biosystems™. The results were analysed in GeneMapper ID v3.2 software.

### Gene expression analysis

#### Inclusion and exclusion criteria

This is a case‒control study in which the sample used in the study consisted of 23 individuals divided into two groups: a control group of 20 individuals without the disease between 18 and 32 years of age and an MPS VII group that consisted of one patient with the new variant and his parents (heterozygotes). The individuals selected for the control group were chosen based on the absence of chronic diseases that could affect the results of the gene expression test. Only individuals who were healthy and without any preexisting diseases were included in the study; those who were born with a disease were excluded. Furthermore, the inclusion of the patient’s parents was due to the patient’s heterozygous genotype for comparison. Expression analyses for different patients were not included due to technical and geographical limitations in obtaining nondegraded samples.

#### Genomic sequencing

The MPS Brazil network carries out next-generation genomic sequencing tests in cases of suspected MPS. Genomic sequencing was performed on peripheral blood samples collected from the parents and the patient with MPS VII with a novel variant. Sequencing was performed on the Ion Torrent Personal Genome Machine using a customized panel with the *GUSB* gene. Brusius-Facchin et al. [17] designed the protocol for the workflow diagnosis using next-generation sequencing (NGS), which was applied to identify the variants in this study in partnership with the MPS Brazil Network. The data were analysed using the Ion Torrent Suite and Ion Reporter bioinformatics platforms, version 5.10.2.0, from Thermo Scientific.

#### RNA extraction and quantification

The peripheral blood of the 23 individuals was collected, and the RNA was extracted using TRI Reagent® Solution from Applied Biosystems™ according to the protocol established by the manufacturer. RNase inhibitors (external and internal) were used in the experiment to avoid RNA degradation. The concentration of the extracted RNA was measured by spectrophotometry using a SpectraMAX i3, which is also capable of determining the purity of the extracted RNA using a standard protocol established for this type of sample and procedure.

#### Complementary DNA (cDNA) and real-time PCR

The extracted RNA was converted into cDNA using a high-capacity cDNA reverse transcription® kit from Applied Biosystems™. The mixture per reaction was composed of 3.7 µL of nuclease-free H_2_O, 2.0 µL of 10 × RT buffer, 2.0 µL of 10X RT Random Primers, 0.8 µL of 25X dNTP Mix (100 mM), 0.5 µL of RNase Inhibitor, 1.0 µL of MultiScribe™ RT and 10 µL of total RNA. The reverse transcription reaction was subsequently performed according to the manufacturer’s manual.

Quantitative real-time polymerase chain reaction by reverse transcriptase (RT‒qPCR) was the selected method because it allows for the visualization, analysis, and quantification of gene expression using software. To perform this analysis, a TaqMan® fluorogenic probe was used.

The genes encoding glyceraldehyde-3-phosphate dehydrogenase (*GAPDH*, probe ID: Hs02758991), beta-actin (*ACTB*, probe ID: Hs00357333), and ubiquitin C (*UBC*, probe ID: Hs00824723) were used as references for evaluating the expression of the analysed pathological gene *GUSB* (probe ID: Hs00939627).

For qPCR, we mixed 5 µL of Applied Biosystems™ TaqMan® Gene Expression Master Mix, 3.5 µL of nuclease-free water, and 0.5 µL of TaqMan probe for each gene. We added 1 µL of a cDNA sample to each well of a 96-well plate and measured it using the Applied Biosystems 7300 Real-Time PCR System.

Data obtained in triplicate from the amplification of the cDNA samples were analysed using NormFinder, geNorm, and BestKeeper software to verify which of the reference genes presented less variation within the tested group.

The method used to analyse the relative expression of the *GUSB* gene was that proposed by Schmittgen and Livak [18]. Relative expression was calculated from the cycle threshold (CT) using the 2^−ΔCT^ method. The average values were calculated from the replicates for each sample, along with their respective standard deviation, based on the cycle threshold (CT). Each sample was normalized by subtracting the mean CT from the respective endogenous control, resulting in the normalized CT (nCT = sample CT − endogenous control CT). To perform the analysis, it was necessary to calculate 2^(−ΔCT)^ and the fold change between samples with different molecular characteristics.

### In silico and structural analysis of the GUSB protein

The following bioinformatics tools (Table 2) were used to evaluate the effects of the new variant p.Leu292Pro and the common variant p.Leu176Phe on the function of the GUSB protein: PolyPhen (polymorphism phenotyping), SIFT (sorting intolerant from tolerant), PROVEAN (protein variation effect analyser), Mutation Taster, and HOPE (Project Have yOur Protein Explained).

**Table 2:** Bioinformatics tools used for the prediction of potential pathogenic effects of protein-coding variants

Name	Main algorithm	Input data
PolyPhen	Provides information on the substitution site, phylogenetic and structural effects of the nsSNP	FASTA protein sequence, mutation
SIFT	Selects relevant proteins and align to determine the conservation level	FASTA protein sequence, mutation
PROVEAN	Measures the difference in sequence similarity before and after a mutation, based on alignment	UniProt ID, mutation
Mutation Taster	Evaluates variant pathogenicity using multiple databases, disease associations, homology modelling, and protein features	Gene identification, Ensembl transcript id, nucleotides around the mutation
HOPE	Analyses point mutations using BLAST, 3D structure calculations, UniProt annotation, and homology modelling	FASTA protein sequence, mutation

GUSB protein sequences from various species were obtained using the Ensembl platform (http://www.ensembl.org) to assess the protein’s conservation of amino acid residues, specifically where the studied variants occur. These sequences were then aligned using the ClustalW program.

### Biochemical analysis

#### Assay for quantification of the enzyme β-glucuronidase in the blood (plasma and leukocytes)

The patient with MPS VII and his parents had their peripheral blood (10 mL) samples collected through venipuncture. After collection, the blood was homogenized with heparin anticoagulant and stored at 4 °C. From the total volume of blood collected, 3 mL was removed for plasma separation through centrifugation at 3000 rpm for 5 min. The plasma was subsequently removed and stored at − 20 °C for later analysis. The method used for leukocyte separation was described by Skoog and Beck [19]. The protein concentration was determined according to the methodology described by Lowry et al. [20].

The fluorometric method described by Beaudet et al. [21] was used to analyse the enzymatic activity of the GUSB enzyme in plasma and leukocytes using a spectrophotometer. It was not possible to obtain leukocytes from the parents’ blood samples. A total of two analyses were performed in duplicate for each sample to quantify the protein in the plasma from the patient with MPS VII who had the new variant and his parents.

#### Quantification of urinary glycosaminoglycans

Creatinine measurement in urine is an initial procedure before the separation and quantification of GAG, which aims to estimate the amount of solute in the urine sample as a function of the excretion of the creatinine metabolite [22]. The method used to measure urinary GAGs involves spectrophotometry and was described by de Jong et al. [23]. This methodology measures the absorbance of the reaction obtained between a buffered solution of dimethylene blue (DMB) and the GAG present in urine. The results are expressed in mg of GAG/mmol of creatinine.

### Statistical analysis

We calculated a Z-score to analysis the differential expression of the *GUSB* gene in patient with MPS VII and group control. The Shapiro–Wilk test was used in this study to assess the normality of the samples. The statistical test used in the study was the Kruskal‒Wallis test with Dunn’s posttest for independent samples to compare the number of cDNA amplification cycles between groups. We used the Kruskal‒Wallis test to compare amplification cycles between parents and the patient with MPS VII in terms of compound heterozygosity. A posttest was used to correct the results for multiple tests. For the analysis of genomic ancestry, the Kruskal‒Wallis test was used. Subsequently, Dunn’s post hoc test was used to verify the difference between pairs, indicating the *p* value and the adjusted *p* value. The tests were two-tailed and *p* value ≤ 0.05 was considered statistically significant.

## Results

### Ancestry markers in patients with MPS VII

**Fig. 2:**
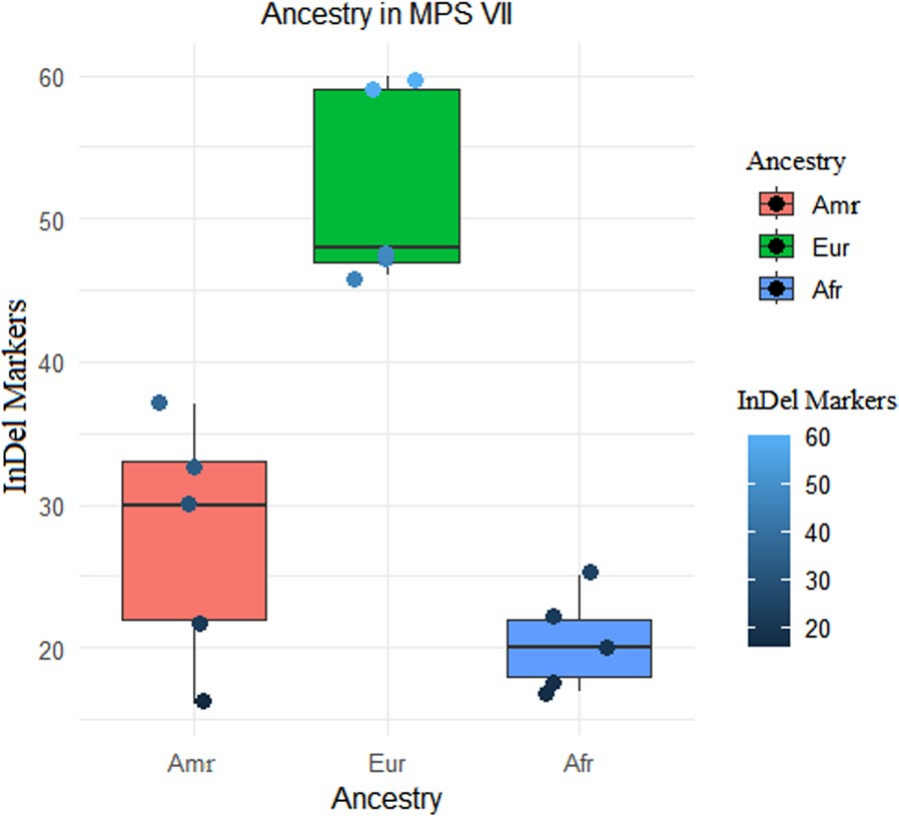
Boxplot comparing Amerindian (Amr), African (Afr) and European (Eur) markers in patients with MPS VII. Significance was determined by the Kruskal‒Wallis test and Dunn’s post hoc test

The distribution of ancestry markers tested in the five patients indicated a significant difference between European and African ancestry (*p* < 0.05). However, there was no significant difference between Amerindian and European ancestry (*p* = 0.07) or between Amerindian and African ancestry (*p* = 0.59), as shown in Fig. 2.

**Fig. 3:**
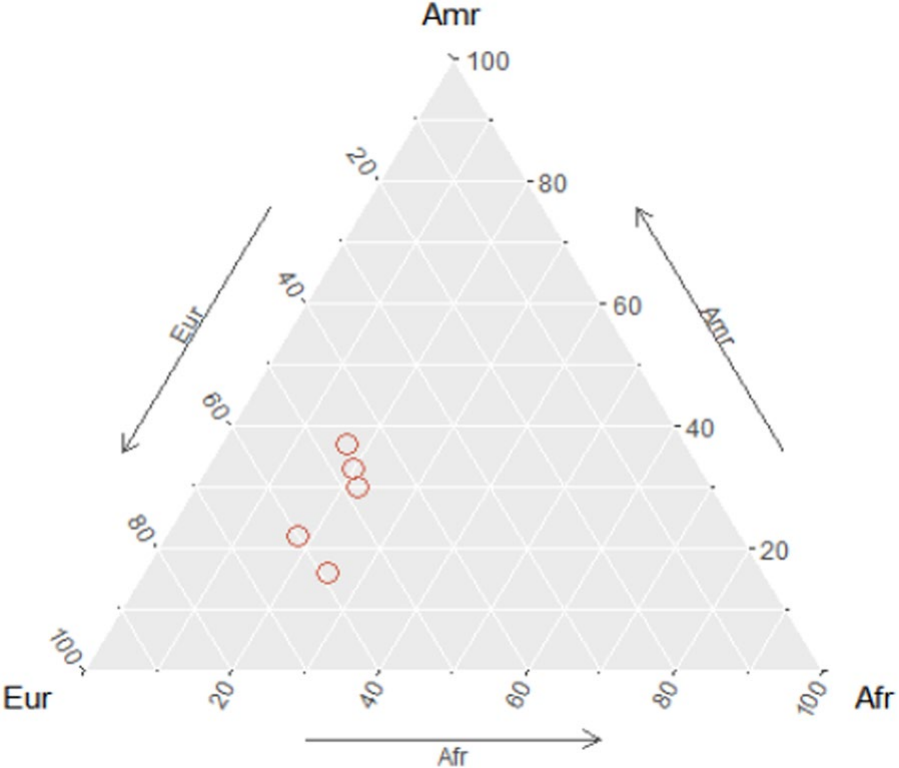
Estimates of the genetic admixture of patients with mucopolysaccharidosis type VII. The diagram displays patients as circles and uses arrows to indicate the most significant ancestry, with points closer to vertices representing a greater contribution of that ancestry

Figure 3 shows the ancestry profile for each patient. The closer one of the circles is to the vertices of the triangle, the greater the ancestry indicated for that patient. This analysis allows visual representation of the different ancestries for this sample group of patients with MPS VII. Notably, the samples tended to be in the lower part of the triangle on the left, indicating European ancestry.

### Quantification of β-glucuronidase enzymes (plasma and leukocytes) and GAGs (urine)

The patient with MPS VII had a plasma β-glucuronidase enzyme activity of 0.70 nmol/h/mL, whereas the activity levels for his father and mother were 43.49 nmol/h/mL and 14.56 nmol/h/mL, respectively (reference value: 30–300 nmol/h/mL). Enzymatic activity in leukocytes was only detected in the patient with MPS VII, resulting in 0.90 nmol/h/mg protein (reference value 23–151 nmol/h/mg protein). The quantification of urinary GAGs in the patient with MPS VII was 68.7 mg/mmol of creatinine (reference value under 5 years: 15.19–51.95 mg/mmol of creatinine).

### Genomic sequencing results

Sequencing analysis revealed that the father was the carrier of the common p.Leu176Phe variant, whereas the mother carried the new variant (p.Leu292Pro). The patient with MPS VII was heterozygous for both variants.

### Analysis of the effects of variants on the expression of the *GUSB* gene

**Fig. 4:**
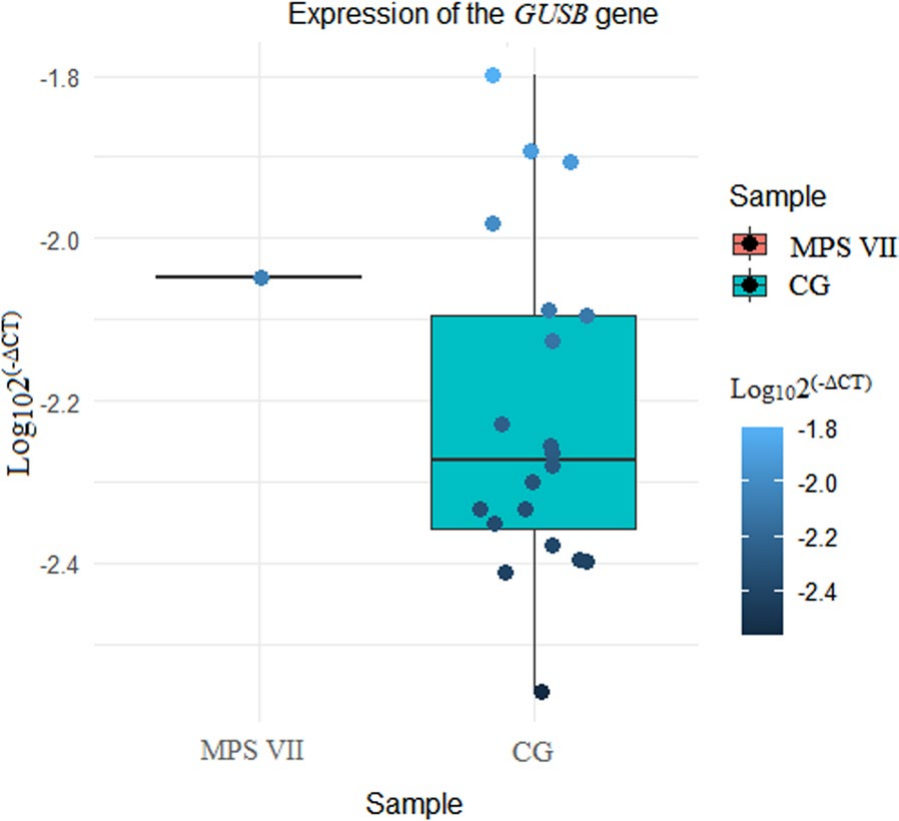
Differential expression of the *GUSB* gene by RT‒qPCR. Quantification is based on Ct values (PCR cycles) and normalized to the reference genes *UBC* and *ACTB* as reference controls. 2^(−ΔCT)^ is the mean of triplicates from 21 different individuals. A. Quantification values on the logarithmic scale for 2^(−ΔCT)^ in a box plot. MPS VII (mucopolysaccharidosis type VII); CG (control group)

The data obtained by RT‒qPCR analysis of the cDNA levels of the *GUSB* gene in both groups indicated that the patient with MPS VII had a higher level of expression (_Log10_2^(−ΔCT)^ = − 2.04822) than did the control group, which presented a mean differential expression of − 2.219019 and a standard deviation of 0.2 (Fig. 4A). Each dot represents one of the 21 samples.

We used a z score to analyse the data and locate the patient with the new variant on the control group scale for differential expression. Compared with the median GC group and MPS VII, the value for the z score was 0.88, with a *p* value = 0.3.

The patient with MPS VII had an expression value that exceeded the first quartile (− 2.358) and was above the second (− 2.094) quartile values.

Darker circles indicate lower-level expression, whereas light blue circles indicate high-level expression. The expression of *GUSB* in the patient with MPS VII was 0.923 times greater than that observed in the control group [− 2.04822/(− 2.219019)].

Analysis of the samples was performed in triplicate in qPCR amplification cycles of the *GUSB* gene. The study compared the control group with the patient with MPS VII by one-way ANOVA with a post hoc Bonferroni correction. Only results compared with those of the patient with MPS VII were considered to elucidate divergences in the amplification cycle in these variants compared with normal genotypes.

**Fig. 5:**
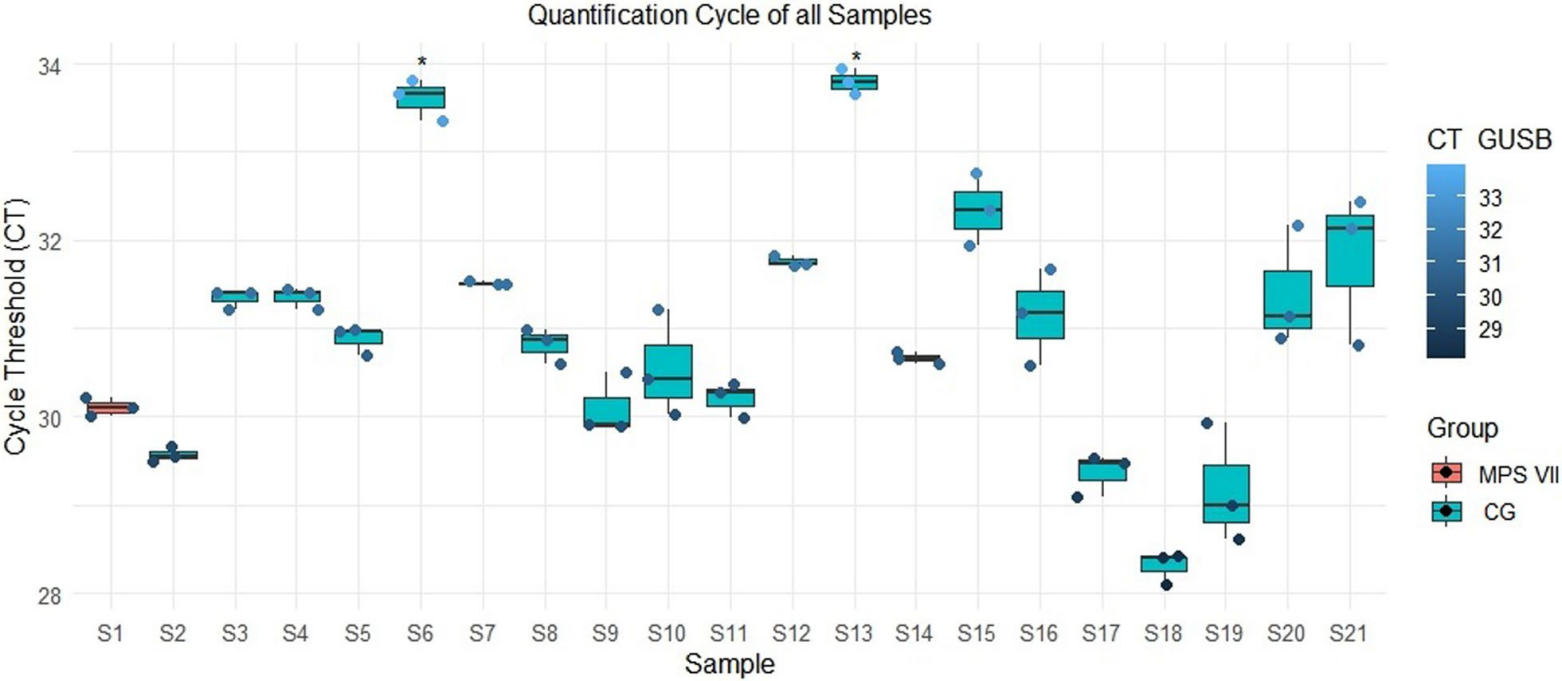
Quantification of the *GUSB* gene by RT‒qPCR. The box plot shows the triplicates of each amplification cycle in 21 samples. The lower the CT is, the higher the fluorescence emission quantified by real-time PCR. The symbol * indicates which sample has *p* < 0.5. MPS VII (mucopolysaccharidosis type VII); CG (control group)

Among the boxplots shown in Fig. 5, 21 samples are represented with their respective triplicates and the threshold of the amplification cycle (CT) on the y-axis. Compared with the patient with mucopolysaccharidosis type VII, 2 individuals showed significantly different values (*p* < 0.05). The other eighteen samples from the group without the disease did not significantly differ.

The patient with MPS VII had a lower amplification cycle value than did nine of the other samples (S3, S4, S6, S7, S12, S13, S15, S20, and S21) but had a greater amplification cycle value than did S18.

**Fig. 6:**
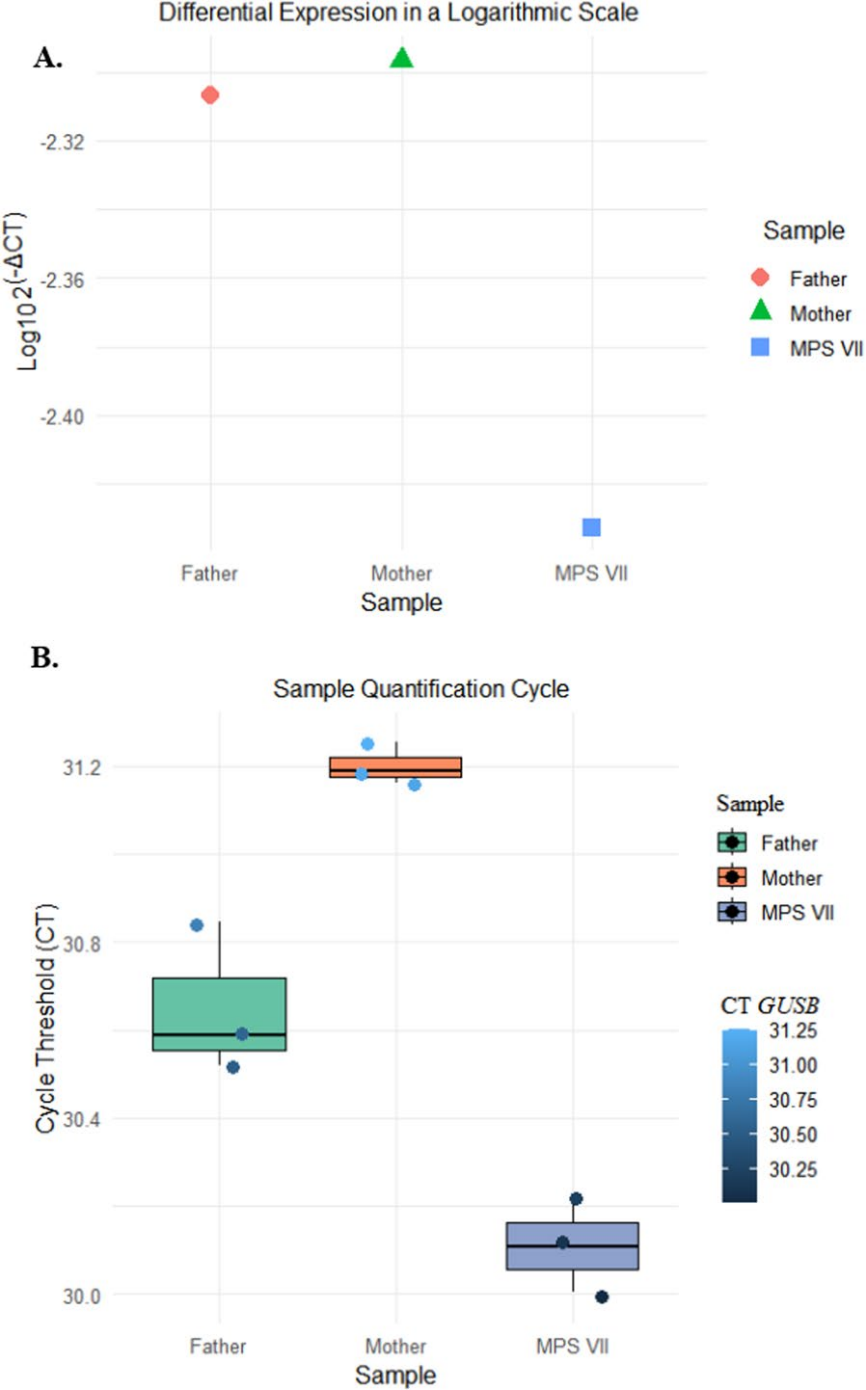
Cycles of quantification of the *GUSB* gene by RT‒qPCR, comparing the patient with MPS VII patient and their father and mother. **A** Differential expression on a logarithmic scale of the 2^(−ΔCT)^ values. The most suitable reference genes used for this analysis were *ACTB* and *GAPDH*. **B** Analysis of triplicates of each application for each sample

A comparison of the gene expression analysis of the parents with that of the patient with MPS VII was successful in this study because of information available on their genotypes. For this step, to normalize the *GUSB* gene, the reference genes chosen were *ACTB* and *GAPDH*, which proved to be more suitable for this type of analysis. The samples were observed in triplicate (Fig. 6).

The data obtained for the *GUSB* gene by RT‒qPCR revealed that the patient with MPS VII presented lower expression than did his parents, with _Log10_2^(−ΔCT)^ values of − 2.4328 for the patient, − 2.2965 for the mother and − 2.3066 for the father (Fig. 6A). Additionally, individual parent/child analysis was performed concerning the number of qPCR amplification cycles.

Figure 6B shows a significant difference (*p* = 0.021) between the mean CT values of the mother (31.201) and the patient with MPS VII (30.109) using the Kruskal‒Wallis test and Dunn’s post hoc test. No significant difference (*p* > 0.05) was found between the CT values of the father and the patient with the new variant. The degree of gene amplification did not significantly differ between the parents (*p* > 0.05), with CT values of 31.201 for the mother and 30.651 for the father.

### New variant characterization

To carry out the in silico prediction of the pathogenicity of the variant, the online tools Polyphen, PROVEAN, HOPE, Mutation Taster, and SIFT were used, which determined that the variant was deleterious or possibly deleterious (Table 3).

**Table 3:** Variants present (compound heterozygosity) in the *GUSB* gene of the patient with MPS VII

Variant (cDNA)	Variant (protein)	Exon	Prediction the effect of the variant on the protein				
			PolyPhen	PROVEAN	SIFT	HOPE	Mutation Taster
c.526C>T	p.Leu176Phe	3	Deleterious	NP	Deleterious^a^	Deleterious	Deleterious
c.875T>C	p.Leu292Pro	5	Deleterious	Deleterious	Possibly deleterious	Possibly deleterious	Deleterious

NP, Not performed

^a^Reference KHAN et al. [24]

## Discussion

### Genetic ancestry in MPS VII

The first case diagnosis of a patient with MPS VII in northern Brazil was in 2018. There are few reports of this disease in other countries, and no more than 20 cases have been reported in Brazil [25]. MPS VII is a rare metabolic disorder. Therefore, a shared genotype referring to the most frequent variant (p.Leu176Phe) in the same country is an unusual case. Knowledge about population ancestry has been considered an important tool to aid in the application of pharmacogenomics [26] and genomic medicine [27]. These themes contribute to the elucidation of existing differences in the clinical treatment of certain diseases.

In this study, patients with MPS VII from various regions showed a significant European genetic contribution, with a secondary influence from Amerindian ancestry. The results indicate that the patients with MPS VII (n = 5) presented a 52% contribution related to European ancestry, differing only statistically from African ancestry (*p* < 0.05). If a larger number of patients had been analysed, the differences in ancestry contributions could have been more accurately estimated. The compound heterozygous patient had the highest European ancestry (60%), followed by Amerindian ancestry (22%) and African ancestry (18%).

The high contribution of European genetic markers to the Brazilian population has already been indicated in some studies [14, 15, 28]. However, there are proportionate differences in the number by region in Brazil, with a higher proportion in South China and Southeast China (77.7% and 73.7%, respectively); in North China and Northeast China, studies have indicated contributions of 69.7% and 60.06%, respectively, for markers of European origin [29]. Studies related to the Brazilian distribution also agree on the genomic predominance of Amerindians in North China and Africans in Northeast China [30, 31].

The initial hypothesis considered in the design of this study is related to the predominance of the p.Leu176Phe variant in the cohort of patients with MPS VII in Brazil. The most prominent European ancestral contribution was possibly more determinant for this group; this variant originated from a founder effect caused by the migration of the Portuguese population or other European populations in one of the settlement events of the country.

Another hypothesis is related to the migration of populations from America. Mexico presents a high number of cases of MPS VII, as highlighted by Mendoza-Ruvalcaba et al. [24]. Thus, there is a possibility that some populations have contributed to the spread of the variant in Brazil, as there were migration events from North America to South America, and the Mexican population represents an ancestral contribution of Amerindians and Europeans [26].

In addition, it is necessary to consider some historical issues in the formation of the cities where patients with MPS VII were born. Figure 1 shows that two cities in Bahia, Araci and Tucano, are located close to each other. This proximity raises the question of the potential social interactions of these places in recent centuries and, consequently, the perpetuation of the p.Leu176Phe variant. Giugliani et al. [10] emphasized that the highest number of MPS VII cases were found in the Northeast Region, specifically in Bahia (n = 5): one in Monte Santo, two in Araci, and two in Tucano. Except for Araci, where two brothers with MPS VII were confirmed in the same family, there are questions about the possible common origin connecting these locations to the incidence of the disease and whether the frequency of the pathogenic variant is higher than what is assumed in Bahia.

A similar situation has been reported with evidence of Iberian origin for one of the rarest variants (p.Ser341Arg) among patients with MPS IVA in the Northeast Region of Brazil [32]. However, the cohort of patients with MPS VII in the Iberian Peninsula presents more diverse pathogenic variants than the one predominantly found in Brazil [33].

Brazil is an admixture country in terms of its ethnic composition; therefore, it is possible that a founding mutation occurred that was dispersed, causing the appearance of this same variant in different regions of the country. Even if this common mutation among patients originated from the emergence of independent mutations, a situation such as this is unlikely given the need for this event to occur individually several times in different locations and owing to the conservative state of the sequence region of amino acids where the p.Leu176Phe variant is found [34].

### *GUSB* expression and in silico prediction of the new variant

Considering the p.Leu292Pro variant, the patient with MPS VII presented more prominent differential expression than did the control group in most samples (Fig. 4). The analysis of the z scores revealed that the expression of the *GUSB* gene in the patient was higher than the average expression level in the control group but not significantly different. This analysis suggested that the slightly lower expression level was not abnormal compared with that in the control group and fell within the normal range of variation. As observed in Fig. 5, the comparison between control and the patient with MPS VII not revealed amplification cycle value different for most of samples in GC. There is a lack of *GUSB* expression on MPS VII available in the literature. This study enhances our understanding of the pathology.

As illustrated in Fig. 4, the fold change suggests that GUSB has a relatively high expression of 0.923 fold for the patient compared with the control group. This result does not reflect the increase in activity of the β-glucuronidase enzyme, as it was lower in the patient: the plasma activity was 0.7 nmol/h/mg (reference value 30–300 nmol/h/mL), and the activity in leukocytes was 0.9 nmol/h/mg protein (reference value 23–151 nmol/h/mg protein). These findings are new in the literature.

Twins with MPS VII, homozygous for p.Leu176Phe, as observed by Wu et al. [35], presented a situation similar to that described in this study. In this case, cell lines with this variant presented low β-glucuronidase activity and expressed the same amount of enzymatic protein as the wild-type cell lines did. For those strains whose mutant protein expression levels were four times greater than those of the wild-type strain, the β-glucuronidase enzymatic activity was greater than that of the wild-type strain in the early stages and decreased over time. According to the results of the present study, the occurrence of overexpression is due to a nonpathogenic variant (p.Pro649Leu) that partially activates a new site in the β-glucuronidase enzyme, thus correcting the deficient activity of the protein, which is at least partly caused by missense variants in MPS VII.

The modifications caused by the p.Leu176Phe variant in the β-glucuronidase enzyme and other variants of MPS VII have already been described by Khan et al. [34]. These alterations cause changes in stability in a very conserved region of the protein due to the increase in the strength of hydrogen bonds. However, the actual cause of the increased expression has yet to be clarified. This relationship between the *GUSB* gene mRNA level and β-glucuronidase activity may be due to posttranscriptional regulation, as noted by the polymorphism in the study by Wu et al. [35].

In terms of individual analyses, the samples from the patient with MPS VII contained significantly more cDNA than the other nine samples did. This is indicated by the lower amplification value, which suggests that a considerable amount of cDNA is present in the sample. Other studies have suggested increased expression of the *GUSB* gene for certain diseases, such as inflammatory and liver diseases and some types of cancer, and as a biomarker for changes in memory in Alzheimer’s disease [36–39].

Moreover, eighteen samples from the control group presented amplification values that were not significantly different from those of the patient with MPS VII for the new variant (Fig. 5). This situation could suggest no difference in expression between groups, and even though the patient with compound heterozygosity showed the phenotypic change manifesting MPS VII, this does not reflect the expression of the *GUSB* gene. To determine whether the p.Leu292Pro variant is the cause of the similarity observed in this study, it is necessary to compare patients with the same MPS VII variant (p.Leu175Phe).

The study also evaluated the parents of the patient, who had normal phenotypes but who were carriers of variants, to compare the expression of the *GUSB* gene because of a methodological obstacle mentioned at the beginning of this study. Given the similarity between the amplification cycles of the father and the patient with MPS VII, it is possible to assume that the p.Leu176Phe variant, present in the father, interferes less with the expression of *GUSB* gene transcripts than the new variant (p.Leu292Pro) present in the mother of the patient. In this case, the gene expression of the cDNA was lower in the mother than in either the patient or his father (Fig. 6B).

The assay of the beta-glucuronidase protein in the patient’s parents and the respective variants present in each one revealed interesting data. The patient’s mother (p.Leu292Pro) had beta-glucuronidase activity of approximately 14.56 nmol/h/mL, which was below the reference value for this enzyme. Conversely, the patient’s father (p.Leu176Phe) had an enzyme activity of 43.5 nmol/h/mL. These results suggest that of the variants possibly interferes more with gene expression than the other and, consequently, with the protein phenotype.

The in silico study of the new mutation revealed that the amino acid residue resulting from the mutation is shorter than that of the wild type and that the variant is located in a region of the protein conserved among many species, such as *Xenopus tropicalis* (frog) and *Danio rerio* (zebrafish) (PolyPhen-2, accessed April 8, 2024), as well as other species. Thus, the substitution for proline in the protein sequence may be responsible for a more moderate MPS VII phenotype, as it modifies the interaction with the protein domains.

**Fig. 7:**
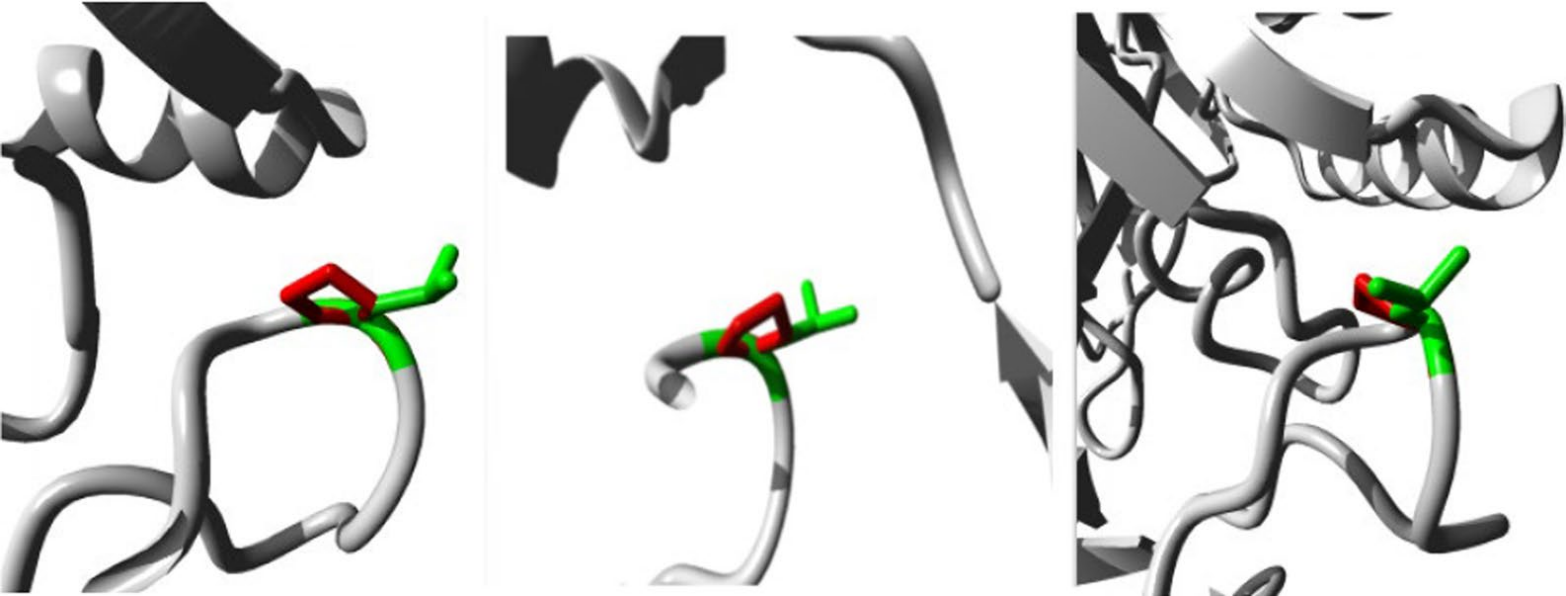
Three-dimensional structure corresponding to the region of the β-glucuronidase where the amino acid residues are located. The figure shows the wild-type residue and the modified variant in green and red, respectively, at different angles. Models were extracted from the HOPE tool

**Fig. 8:**
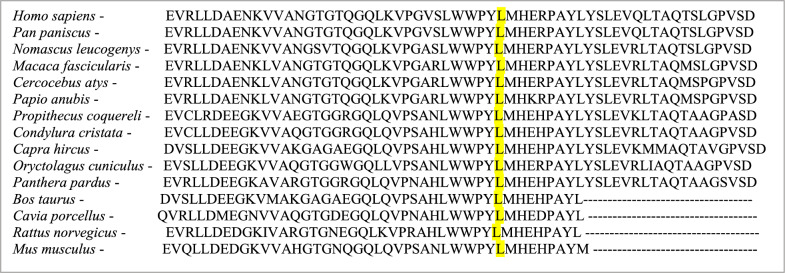
Alignment of several species of the β-glucuronide protein near amino acid L.292

The modifications caused by the replacement of the leucine residue with a proline at position 292 allow new polar and nonpolar alterations in nearby structures, such as those of 293Met (Fig. 7). New chemical interactions could reduce the enzyme’s affinity for the substrate or its ability to catalytic activity. Furthermore, this substitution can modify interactions with protein domains, such as those in the beta-galactosidase/glucuronidase superfamily. The regions associated with these amino acid residues are highly conserved among organisms (Fig. 8), and the amino acid proline was not observed among the other species studied in this research.

Khan et al. [33] conducted research to clarify the pathogenic potential of the variants in the protein that cause MPS VII. Among the in silico analyses carried out, p.Phe208Pro, p.Phe539Gly, and p.Leu622Gly were reported to destabilize the protein and change its structure. Variants such as p.Glu37Phe and p.Ser71Leu were predicted to be mutations that increase stability, which would prevent changes in the protein’s conformation for its typical performance. It is necessary to determine how the new variant p.Leu292Pro described in this study could affect protein stability and performance.

Our study is a continuation of previous work that identified a new variant in the *GUSB* gene in a patient with MPS VII [10]. This study is a pioneer in evaluating how ancestry can be an essential parameter to define the origin of variants in the *GUSB* gene in patients with MPS VII in Brazil. There are several ways of interpreting the pathogenicity of a new variant of MPS, provided that there are adequate resources for the analyses. Determining the frequency of the variant in a representative amount of the population would be an initial alternative, although this approach would not explain the damage to the protein structure. In silico studies are used as a strategy to predict how much the substitution of leucine for proline can impair the three-dimensional model and the kinetic and structural properties of β-glucuronidase despite not informing the degree of pathogenicity of the variant. Thus, expression studies are necessary to understand how transcripts can confer greater or lesser stability to the enzyme in question. The next step would be to use in vitro models to evaluate how the new variant p.Leu292Pro interferes with the activity of β-glucuronidase. A group of these tools would be appropriate for providing a better understanding of the genotype and phenotype relationships in patients with MPS VII, especially in Brazil.

## Conclusions

Ancestry analysis using INDEL markers suggested European ancestry for the origin of MPS VII, with the greatest contributions for children with compound heterozygosity. However, it is essential to note that the genomic ancestry of a population is complex, given the levels of miscegenation over the centuries among diverse ethnicities, be they Amerindian, European, African, or others. There are still limitations in studies of ancestry due to the grouping of ethnicities that seem similar but may differ in some respects. Evolutionary features and geographic barriers of group populations in places with more considerable geographic extensions have influenced these issues. Examples include the African continent and South America. This study is significant because it is the first to investigate the ancestry of MPS VII and to elucidate the higher incidence of the disease in Brazil.

Gene expression analysis of MPS VII variants in the *GUSB* gene revealed similar mRNA expression compared with that in the control group. The data on gene expression differed from the data obtained from the protein dosage of the β-glucuronidase enzyme compared with parents with normal phenotypes when we analysed the gene expression and protein expression data, indicating the existence of complex mechanisms in transcriptional and posttranscriptional processing. The p.Leu292Pro variant was found to be deleterious in silico. Further studies need to be performed to understand how the same genotype of Brazilian patients with MPS VII (with the exception of patients from the North Region of Brazil) may explain the considerable clinical heterogeneity observed in MPS VII.

## Data Availability

The experimental data underlying the findings of this study are accessible on Figshare with the identifier 10.6084/m9.figshare.27708315.v2 [40].

## Abbreviations

MPS VII: Mucopolysaccharidosis type VII
GAG: Glycosaminoglycans
DS: Dermatan sulfate
HS: Heparan sulfate
C4S: Chondroitin 4-sulfate
C6S: Chondroitin 6-sulfate
*GUSB*: Beta-glucuronidase
INDEL: Insertion or deletion
HCPA: Hospital de Clínicas de Porto Alegre
cDNA: Complementary DNA
NGS: Next generation sequencing
Polyphen: Polymorphism phenotyping
SIFT: Sorting intolerant from tolerant
PROVEAN: Protein variation effect analyser
HOPE: Have yOur Protein Explainede
AMR: Amerindian
EUR: European
AFR: African
GAPDH: Glyceraldehyde-3-phosphate dehydrogenase
ACTB: Beta-actin
UBC: Ubiquitin C
